# Increased Carotid Thickness in Subjects with Recently-Diagnosed Diabetes from Rural Cameroon

**DOI:** 10.1371/journal.pone.0041316

**Published:** 2012-08-20

**Authors:** Nicola Napoli, Enrico Zardi, Rocky Strollo, Michele Arigliani, Andrea Daverio, Flaminia Olearo, Daniele Tosi, Giordano Dicuonzo, Filomena Scarpa, Claudio Pedone, Hervé Hilaire Tegue Simo, Giovanni Mottini, Paolo Pozzilli

**Affiliations:** 1 Endocrinology & Diabetes, Campus Bio-Medico University of Rome, Rome, Italy; 2 Division of Bone and Mineral Diseases, Washington University School of Medicine, St. Louis, Missouri, United States of America; 3 Clinical Medicine, Immunology and Rheumatology, Campus Bio-Medico University of Rome, Rome, Italy; 4 Laboratory of Clinical Microbiology, Campus Bio-Medico University of Rome, Rome, Italy; 5 Geriatrics, Campus Bio-Medico University of Rome, Rome, Italy; 6 Ministry of Health District of Baham, Baham, Cameroon; 7 Centre for Diabetes, Barts and The London School of Medicine, Queen Mary University of London, London, United Kingdom; Universita Magna-Graecia di Catanzaro, Italy

## Abstract

**Background:**

We have recently shown a high prevalence of diabetes and obesity in rural Cameroon, despite an improved lifestyle. Diabetes in rural Africa remains underdiagnosed and its role in increasing risk of atherosclerosis in these populations is unknown. We investigated the prevalence of carotid atherosclerosis and cardiovascular risk factors in a population of subjects with recently-diagnosed diabetes from rural Cameroon.

**Methodology/Principal Findings:**

In a case-control study, carotid intima-media thickness (IMT) was measured in 74 subjects with diabetes (diagnosed <2 years), aged 47–85 and 109 controls comparable for age and sex. Subjects were recruited during a health campaign conducted in April 2009. Blood glucose control (HbA1c, fasting blood glucose) and major cardiovascular risk factors (complete lipid panel, blood pressure) were also measured. Mean carotid IMT was higher in subjects with diabetes than healthy controls at each scanned segment (common, internal carotid and bulb) (*P*<0.05), except the near wall of the left bulb. Vascular stiffness tended to be higher and pressure-strain elastic modulus of the left carotid was increased in subjects with diabetes than controls (*P*<0.05), but distensibility was similar between the two groups. At least one plaque >0.9 mm was found in 4%, 45.9% and 20% of diabetic subjects at the common, bulb or internal carotid, respectively. Only 25% of patients had an HbA1c<7%, while over 41.6% presented with marked hyperglycemia (HbA1c>9%). The prevalence of diabetic subjects with abnormal levels of LDL-cholesterol, triglycerides, HDL-cholesterol or blood pressure was 45%, 16.6%, 15% and 65.7%, respectively.

**Conclusions:**

Carotid thickness is increased in subjects with diabetes from a rural area of Cameroon, despite the relatively recent diagnosis. These findings and the high rate of uncontrolled diabetes in this population support the increasing concern of diabetes and cardiovascular diseases in African countries and indicate the need for multifaceted health interventions in urban and rural settings.

## Introduction

It has been reported that by 2030, there will be more than 228 million diabetic patients in developing countries, twice as many as in the western nations, with the highest rates in the sub-Saharan region [1].

However, diabetes in African countries has been neglected for years, and only recently has it risen to the attention of the scientific community [2], [3], [4]. Because of the dearth of published clinical studies, it is believed that the prevalence of diabetes may be underestimated in African countries, and therefore many patients with diabetes are left untreated.

Paradoxically, cardiovascular disease accounts for a higher rate of mortality in developing countries than in developed ones [5], highlighting the importance of diabetes complications in terms of mortality and morbidity [6], [7].

Poor knowledge by the patients about the condition and limited access to healthcare, the diagnosis is often made at a late stage of the disease, when infections, retinopathy, and vascular complications have already caused irreversible damages [8], [9], [10]. For this reason, the Medical Research Council of South Africa has developed a national research program focused on cardiovascular diseases, indicated as “public enemy number 2”, after HIV [11].

So far, however, few studies on diabetes prevalence have been conducted. We have recently shown a high prevalence of diabetes and obesity in a rural area of Cameroon, confirming that even in a setting with a protective environment, diabetes is an important and neglected health problem [12]. In fact, most patients were unaware of having diabetes and none was on a regular treatment. We report on a clinical survey in the same rural area (district of Baham, North Cameroon), about biochemical features and atherosclerosis prevalence in diabetic patients relative to age and sex comparable population.

## Methods

### Participants

Seventy-nine Cameroonian subjects with diabetes and 130 age and sex comparable healthy subjects were studied in the district of Baham, a rural area of Cameroon during a health campaign started in April 2009. Among all these subjects 5 diabetic and 21 controls were excluded from the final analysis because of lack of clinical or biochemical data, therefore we here report data on 74 patients with diabetes and 109 controls. The diabetic population (57% females and 43% males) was in part composed of patients diagnosed during a previous health campaign conducted in 2007 (52 patients) which clinical and biochemical data have been published elsewhere [12]. However, in these patients only capillary glycaemia and anthropometric measures were available. Study subjects were screened through advertisements and announcements by local authorities or referred by local physicians or health providers. After being diagnosed in 2007, patients received diet counseling and assigned to local health providers for follow-up. Nutritional therapy was advised by a certified dietician of our team, using dedicated brochures and showing plates with required food sizes in order to achieve a caloric intake from 1600 to 1400 kcal according to personal needs. Moreover, physical activity was advised as well even if patients were regularly engaged in daily activities which required long walks or other physical tasks (manual labor, agriculture, etc). However, none of those returning at follow-up was on regular treatment or been screened for chronic complications. Other 22 patients included in the study were referred by local physicians as new diagnosed patients. Thus, all diabetic patients included in this study were considered drug-naïve and were diagnosed with diabetes for less than 2-years.

### Clinical data and measurements

All subjects received a physical examination including blood pressure, weight, height and waist and hip measurements. Body mass index (BMI) was calculated (kg/m^2^). Blood glucose was measured using a Glucocard blood glucose analyzer (Lifescan, USA) on capillary blood samples. Blood was drawn after an overnight fast of at least 12 hours to assess glycated hemoglobin (HbA1c), creatinine, urea, AST and ALT, total cholesterol, HDL-cholesterol and triglycerides. Blood samples were sent to and analyzed at University Campus Bio-Medico laboratories in Rome. LDL-cholesterol was calculated using the Friedwal's formula. Physical activity was expressed as a numerical score and was defined as sedentary (score of 1: sitting or lying most of the day), moderately active (score of 2: being on one's feet more than half of a day), and very active (score of 3: engaging in regular physical exercise) [13].

### Carotid ultrasonography

An accurate medical history and physical examination were performed in order to evaluate possible sings of neuropathy and peripheral vascular disease. The carotid intima-media thickness (IMT) was used as morphologic index of macrovascular disease and subclinical atherosclerosis. Carotid IMT is a widely used tool for the assessment of macrovascular risk and can be used to predict coronary heart diseases and stroke [14], [15]. Both common carotid arteries were scanned by the same operator using a portable high-resolution ultrasonographic system (logiq e) equipped with a 5–13 MHz linear transducer. During sonographic examination, all patients were put in the supine position and scanning was performed insonating the vessel in perpendicular orientation, optimazing B-mode settings of gain, depth, focal zone placement, and compression to enhance arterial wall structures and image quality. Measurements were made in on-line mode, where the lumen-intima interface was regular and parallel to the adventitia, using an electronic caliper to calculate the distance between the two echogenic lines that compose the interface of the carotid wall; regions of interest were at the distal-common carotid artery (1 cm before the dilation of the carotid bulb), at the carotid bulb and at the first portion of the internal carotid artery (1 cm after the dilation of the carotid bulb) by a lateral longitudinal projection. Carotid IMT was calculated in these three sites, on the near and the far wall, and the average of six measurements from the right and left side was used for data analysis. The carotid IMT was defined as the distance between the media–adventitia interface and the lumen–intima interface. The same investigator performed all of the ultrasonographic procedures. A value of IMT >0.9 mm was used as index of clinical organ injury [16], and a value of IMT >1.5 mm defined the presence of atherosclerotic plaque [16], [17].

Doppler parameters of common carotid and internal carotid arteries of both sides were also obtained after positioning the sample volume of the ultrasound beam in the middle of the vessel, using an insonation angle of 60° between ultrasound beam and the longitudinal axis of the carotid segment; thus, peak systolic velocity was assessed, and resistivity and pulsatility indices (RI and PI) were automatically calculated by the sonographic machine using the following formulae:
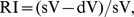
</disp-formula></xsl:text> 

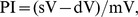
</disp-formula></xsl:text> where sV = peak systolic velocity; dV = end diastolic velocity and mV = mean velocity.


The mean value of four measurements from the right and left side was used for statistical evaluation.

In accordance with others [18], we also used traditional carotid indices of arterial stiffness [vascular strain (VS), vascular distensibility (VD), vascular stiffness (VSf) and pressure-strain elastic modulus (PSEM)] for a better comparison.

VS is the relative change in end diastolic diameter during pulsatile distension.

VD is the relative change in diameter, whereas, VSf is its reciprocal measure; in fact, increasing distensibility implies decreasing stiffness. Finally, PSEM is the pressure change required for (theoretic) 100% increase in diameter

Stiffness parameters were calculated applying the equations shown below, according to the cited references:
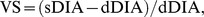
</disp-formula></xsl:text> 

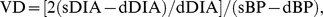
</disp-formula></xsl:text> 

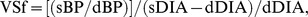
</disp-formula></xsl:text> 

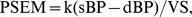
</disp-formula></xsl:text> where sDIA = systolic carotid diameter; dDIA = diastolic carotid diameter; sBP = systolic blood pressure; dBP = diastolic blood pressure and k = conversion factor from mmHg to Pa (k = 7×10^−3^).


These parameters, which have been related to brachial artery flow motion dilation [19], were measured on both common carotid arteries 2 cm proximal to the carotid bifurcation at a plaque-free site by M-Mode ultrasonography with synchronous ECG; after using a minimum pressure on the carotid to allow optimal visualization without compressing the artery, the minimum arterial diameter was assessed between the P-wave and R-wave of the recording ECG whereas, the maximum arterial diameter was assessed between the R-wave and T-wave.

The mean value of four measurements from the right and left side was used for statistical evaluation. Composite IMT were calculated as a mean of IMT from two or three carotid sites, namely mean IMT from internal carotid, common carotid and bulb.

### Informed Consent

The research protocol was approved from both University Campus Bio-Medico Ethical Committee and National Bioethics Committee of Cameroon. Given that most of the population in the rural villages where the study took place is illiterate and speaking different dialects, the National Bioethics Committee of Cameroon approved the use of an oral informed consent. Therefore, informed consent was community-based and was given orally. Awareness of the rural population was obtained by health authorities of the district of Baham and by traditional authorities (Roi de la Chefferie).

### Statistical methods

Results are expressed as mean or proportions with 95% confidence. Group comparisons were made using t-test for continuous variables and by chi-squared analysis for categorical variables (smoking exposure, family history of diabetes, and physical activity). Data were managed using Excel 2003 (Microsoft Corp., Redmond, WA) and were analyzed using R for Linux 2.15 (R Foundation for Statistical Computing, Wien, Austria).

## Results

Clinical and laboratory data of diabetic patients and healthy subjects are presented in Table 1. A subgroup of 49 patients with diabetes were part of a previous survey [12] and were re-evaluated for the present study. In comparison, significant decrease in terms of fasting glucose (226.5±17.5 vs 182.5±12.6, p 0.04) and a slight, not significant reduction in body weight (77.4±3.1 vs 72.1±2.3) was observed. Analyzing the whole population, there were no differences in age, sex, BMI between the two studied groups. Capillary blood glucose and HbA1c were significantly higher in patients with diabetes compared to healthy subjects ones. Among patients with diabetes, 41.6% had marked hyperglycemia (HbA1c>9%) and only 25% achieved a value of HbA1c<7%. ALT levels were higher in patients with diabetes but the average fell into the normal range and had no clinical relevance in all patients. No differences in other clinical or biochemical parameters were found. Among subjects with diabetes, 45% had LDL-cholesterol >100 mg/dL, 16.6% had triglycerides >150 mg/dL, 15% had low HDL-cholesterol (males <40 mg/dL and females <50 mg/dL) and 65.7% had high blood pressure (systolic or diastolic blood pressure >130/85 mmHg, respectively). Patients with diabetes were significantly more active than control subjects. All subjects underwent ultrasonographic examination of carotids (Table 2). Mean IMT (mm) was higher in patients with diabetes than healthy subjects in all examined segments, except at the near wall of the left bulb. Accordingly, composite IMT were significantly higher in subjects with diabetes than controls. Among the diabetic patients 4%, 45.9% and 20% had an IMT >0.9 mm at the common, bulb or internal carotid, respectively. An atherosclerotic plaque with IMT >1.5 mm was found in 1.3%, 13.5% and 12.2% at the common, bulb and internal carotid of subjects with diabetes, respectively.

**Table 1:** Clinical and biochemical features of patients with diabetes and healthy controls.

	Patients with diabetes	Healthy controls	P value
Age, yrs	62.4 (60.2–64.5)	61.3 (59.5–63.1)	0.451
BMI, Kg/m^2^	28.1 (26.3–29.8)	28.6 (27.3–29.9)	0.621
Waist circumference, cm	98.3 (94.2–102.4)	96.4 (93.6–99.2)	0.448
Systolic blood pressure, mmHg	143 (136–150)	138 (133–143)	0.264
Diastolic blood pressure, mmHg	86 (82–90)	84 (81–87)	0.561
Differential pressure, mmHg	57.4 (52.5–62.3)	54 (50.5–57.5)	0.259
Fasting blood glucose, mg/dl	183 (160–209)	88 (83–92)	<0.001
HbA1c, %	9 (7.6–10.3)	6 (5.5–6.4)	<0.001
Total cholesterol, mg/dl	183.5 (172–195.1)	184 (173.4–194.5)	0.954
HDL cholesterol, mg/dl	59.1 (54.9–63.4)	63.6 (59.9–67.3)	0.117
LDL cholesterol, mg/dl	102.2 (93.2–111.3)	101.4 (92.5–108.9)	0.802
Triglycerides, mg/dl	110.5 (97.8–123.3)	98.2 (85.2–111.2)	0.178
Creatinin, mg/dl	1 (0.9–1)	1.1 (0.8–1.5)	0.277
AST, IU/l	43 (36–51)	37 (29–44)	0.227
ALT, IU/l	28 (23–33)	21 (19–24)	0.013
Physical activity, %			
Low	50 (39–61)	9 (4–19)	<0.001
Medium	43 (32–55)	88 (78–94)	
High	7 (3–16)	3 (0–10)	
Smoking, %			
No smoke	89 (79–95)	76 (65–85)	<0.053
Less than 20 cig/day	7 (3–16)	11 (5–20)	
More than 20 cig/day	4 (1–12)	13 (7–24)	
Family history for diabetes, %	64 (51–74)	75 (63–84)	0.195

Data are means (95% confidence interval).

**Table 2:** Carotid ultrasound measurements in patients with diabetes and healthy controls.

	Patients with diabetes	Healthy controls	P value
Left common carotid			
IMT near wall, mm	0.64 (0.61–0.68)	0.58 (0.56–0.6)	0.001
IMT far wall, mm	0.66 (0.63–0.7)	0.59 (0.57–0.61)	<0.001
PI	1.66 (1.56–1.77)	1.63 (1.56–1.69)	0.601
RI	0.74 (0.73–0.76)	0.73 (0.72–0.74)	0.225
M mode	0.57 (0.53–0.62)	0.62 (0.58–0.66)	0.106
Vascular strain, mm	0.092 (0.085–0.1)	0.1 (0.09–0.11)	0.111
Vascular distensibility, mmHg^−1^	0.0037 (0.0032–0.0041)	0.0041 (0.0037–0.0045)	0.145
Vascular stiffness	19.6 (17.3–21.9)	16.7 (14.9–18.6)	0.054
PSEM, Pa	4.72 (4.03–5.41)	3.83 (3.34–4.31)	0.037
Right common carotid			
IMT near wall	0.63 (0.6–0.65)	0.59 (0.57–0.61)	0.017
IMT far wall	0.64 (0.61–0.67)	0.6 (0.58–0.62)	0.043
PI	1.69 (1.57–1.8)	1.55 (1.47–1.63)	0.057
RI	0.74 (0.73–0.76)	0.73 (0.71–0.74)	0.082
M mode	0.55 (0.51–0.59)	0.59 (0.55–0.64)	0.166
Vascular strain, mm	0.089 (0.082–0.096)	0.095 (0.088–0.102)	0.200
Vascular distensibility, mmHg^−1^	0.0035 (0.0031–0.0039)	0.00394 (0.00353–0.00434)	0.146
Vascular stiffness	20.3 (17.9–22.7)	18.1 (15.8–20.4)	0.193
PSEM, Pa	4.89 (4.16–5.62)	4.1 (3.55–4.65)	0.087
Left internal carotid			
IMT near wall, mm	0.59 (0.55–0.62)	0.53 (0.52–0.55)	0.005
IMT far wall, mm	0.61 (0.57–0.65)	0.55 (0.52–0.58)	0.032
PI	1.24 (1.14–1.34)	1.17 (1.11–1.22)	0.222
RI	0.65 (0.62–0.68)	0.63 (0.62–0.64)	0.311
Right internal carotid			
IMT near wall, mm	0.59 (0.56–0.62)	0.53 (0.51–0.55)	<0.001
IMT far wall, mm	0.62 (0.57–0.67)	0.55 (0.52–0.58)	0.019
PI	1.32 (1.23–1.42)	1.28 (1.21–1.35)	0.45
RI	0.69 (0.66–0.72)	0.65 (0.63–0.66)	0.021
Bulb, left carotids:			
IMT near wall, mm	0.69 (0.65–0.73)	0.68 (0.65–0.71)	0.042
IMT far wall, mm	0.81 (0.76–0.87)	0.74 (0.7–0.78)	0.036
Bulb, right carotids:			
IMT near wall, mm	0.72 (0.68–0.76)	0.7 (0.66–0.73)	0.449
IMT far wall, mm	0.8 (0.75–0.85)	0.73 (0.7–0.77)	0.037
Composite IMT, mm (Bulb/common carotid)	0.69 (0.66–0.71)	0.63 (0.61–0.65)	<0.001
Composite IMT, mm (Bulb/internal carotid)	0.67 (0.64–0.69)	0.6 (0.59–0.62)	<0.001
Composite IMT, mm (Bulb/common/internal carotid)	0.65 (0.63–0.67)	0.59 (0.58–0.61)	<0.001

Data are mean (95% confidence interval). IMT, Intima-media thickness; PI, pulsatily index; RI, resistive index; PSEM, pressure-strain elastic modulus.

There was no significant difference in the mean RI and PI of common carotid and PI of internal carotid between diabetic patients and healthy subjects. By contrast, RI of the internal carotid was significantly higher in diabetics compared to healthy subjects. Vascular stiffness tended to be higher and PSEM of the left carotid was increased in subjects with diabetes than controls, but distensibility was similar between the two groups (Table 2).

## Discussion

Cardiovascular disease is still the leading cause of death worldwide in patients with diabetes, and it is now becoming an emerging threat for sub-Saharan Africa and other developing countries [20]. In this study we report on increased carotid thickness in a population of patients with recently diagnosed diabetes from rural Cameroon compared with healthy controls. We also find that most diabetic patients had high HbA1c and most of them had marked hyperglycemia.

Carotid IMT is a validated measure of generalized atherosclerosis. A thickened carotid IMT correlates with an increased risk of myocardial infarction and stroke [14], [15], [21] and it is predictive of future events of silent brain ischemia [22] and coronary heart diseases in subjects with type 2 diabetes [23], [24].

Diabetes duration is an important factor impacting on carotid thickening [25], [26], [27], [28]. Liu *et al.* showed that carotid IMT was significantly related with the duration of type 2 diabetes for longer than 2 years [26]. A report by the Insulin Resistance Atherosclerosis Study (IRAS) group on a large multiethnic population demonstrated that internal carotid IMT of patients with diabetes is not different from non-diabetic subjects at the time of diagnosis, but it takes a few years to increase significantly [27], [28]. Interestingly, in our study internal carotid IMT was higher in subjects with diabetes than in healthy controls despite the relatively short time from diagnosis (less than 2 years). A likely explanation for this finding is that diabetes had gone undiagnosed for long time before patients were screened. More than 80% of diabetes cases in Cameroon are inadequately diagnosed, and the percentage is even higher in the rural population [29], almost double the figure compared with Western countries [30]. However, several differences in patients' features and methodology make difficult a full comparison between our study and the IRAS. Although carotid IMT was increased in subjects with diabetes compared with controls, the number of subjects with plaques (IMT>1.5 mm) was low and the maximal mean IMT at the common and internal carotid was generally lower than IMT shown as predictive for cardiovascular events in longitudinal studies [15], [31]. A lower exposure to recognized risk factors in our population may be protective. Unfortunately, studies analyzing the effect of diabetes on sub-clinical atherosclerosis in sub-Saharan Africa are limited [32], [33], [34], and the comparison with our data is biased by differences in methodology and patients' features (type of diabetes, diabetes duration, site of IMT measurement). Moreover, most of these studies on cardiovascular complications are conducted in urban areas or are predominantly on populations of Caucasian ancestry. In a study by Faeh et al. on pre-diabetic and diabetic patients from the Seychelles (predominantly of African ethnicity), common carotid IMT was significantly increased in diabetic patients and showed a substantial progression across the categories of impaired glucose [34]. The difference was similar to that found in our study, although the absence of internal carotid IMT measures (that are much more dependent of the stage of diabetes) limits the comparison. Of note, similarly to our study, less than a fifth of the diabetic patients studied by Faeh et al. met the therapeutic target for blood glucose, blood pressure or blood cholesterol [35].

These data are relevant given that late diagnosis or intervention increase the probability of irreversible organ damage progression. This risk may even be underestimated considering that most of the patients were off of their glycemic target (about 1.6 times higher than that reported in Western countries [36]). Patients were also not seen by physicians or any health providers for diabetes on a regular basis or screened for diabetic complications. Importantly, none of them was on a stable glucose-lowering treatment after the diagnosis. Indeed, prospective intervention studies provided evidence that blood glucose-lowering medications may reduce the IMT progression linked to diabetes even during limited time-frames [37], [38]. More importantly, it is well-known that intensive control of blood glucose prevents 25 to 70% of micro-vascular complications and reduces mortality by 12% [39]. It is also known that if started late after the onset of the disease, therapy loses its beneficial effect on cardiovascular disease [40], [41]. Although health promotion and education programs in Cameroon have been shown to increase the population's awareness to diabetes [42], it is still difficult to ensure the spread of these campaigns over the country.

Several other countries do not even have a diabetes program and only a few countries can afford to screen and treat diabetes complications [43]. Although efforts aimed at enhancing access to medications have been carried out in the last years, the cost of medicines is still unaffordable for most of the population and medical counseling remains extremely low, particularly in the rural context. For example, only 25% of African countries can provide insulin in rural areas, a situation virtually unchanged from 10 years ago [44].

In conclusion, our data obtained from a rural area of sub-Saharan Africa show that carotid thickness is higher in subjects with diabetes than in healthy controls despite the relatively short time from diagnosis, suggesting that the disease remains undiagnosed and untreated for several years. This finding and the high rate of uncontrolled diabetes in this population increases the concern of diabetes in African countries raised and support the need for multifaceted health interventions in urban and rural settings. Considering that the healthcare system in these countries has not adapted to this new reality, well-planned cost-effective strategies of prevention and treatment and refined tools to assess health services and monitor progress are necessary in order to contrast the upcoming epidemic of diabetes-related complications.
